# Roux-en-Y Gastric Bypass and the Clinical Manifestations of Vitamin Deficiencies: Case Report and Literature Review

**DOI:** 10.7759/cureus.56101

**Published:** 2024-03-13

**Authors:** Trystan A Innes, Samuel R Massey, Hezborn M Magacha, Venkata Vedantam, Neethu Vedantam

**Affiliations:** 1 Internal Medicine, Quillen College of Medicine at East Tennessee State University, Johnson City, USA; 2 Infectious Diseases, Quillen College of Medicine at East Tennessee State University, Johnson City, USA

**Keywords:** nutrition, clinical manifestation vitamin deficiencies, roux-en-y gastric bypass (rygb), vitamin deficiencies, roux-en y complication

## Abstract

This case outlines the complications of Roux-en-Y gastric bypass surgery (RYGBS) and demonstrates clinical manifestations of several vitamin deficiencies. We present a 45-year-old female patient who was admitted to our hospital with generalized weakness, anasarca, hypotension, and failure to thrive, a year after she had her RYGBS. After the procedure, she had nausea, vomiting, and diarrhea with progressive inability to tolerate any oral intake. Subsequently, the patient lost over 200 pounds and got bedridden. Initially, after the procedure, the patient had a dilatation of her anastomotic stricture, but after her surgeon moved out of town she was lost to follow up until she presented to our hospital. Upon arrival, the patient was hypotensive, tachycardic, and appeared dehydrated. The exam also revealed several clinical manifestations of vitamin deficiencies including dermatitis concerning Pellagra, follicular hyperkeratosis, and Bitot spots. Laboratory data showed significantly low albumin, protein, acute kidney injury, and several electrolyte abnormalities. The patient had to be admitted to the ICU for pressure support along with colloid and electrolyte replacement. An Esophagogastroduodenoscopy (EGD) was performed which revealed a clean-based ulcer and a 10-mm anastomotic stricture. She was started on Total Parenteral Nutrition (TPN). After the dilatation of the stricture, the patient was able to tolerate oral intake and TPN was subsequently discontinued upon discharge. The patient was educated extensively on the importance of compliance with daily vitamin supplementation and regular follow-up with bariatric physicians at discharge.

## Introduction

The World Health Organization (WHO) has recognized obesity as a global epidemic with many consequences for both the individual and society. Obesity increases the risk for mental health disorders, obstructive sleep apnea, diabetes, hypertension, and coronary artery disease [1]. Furthermore, it is estimated the medical cost of adult obesity in the United States ranges from 147 billion to nearly 210 billion dollars annually [2]. Surgical gastric bypass is often utilized when both lifestyle modifications and pharmaceutical therapy fail to impart meaningful weight loss. Roux-en-Y gastric bypass surgery (RYGBS) is a commonly performed bariatric procedure in which a novel small pouch is constructed from the stomach and anastomosed with the jejunum. It is medically indicated for obese patients with a body mass index (BMI) over 35, BMI 3 with type 2 diabetes, and BMI 30 with unsuccessful attempts at weight loss or other comorbidities. Thus, significant weight loss is imparted, not only because of malabsorption of macronutrients by bypassing the stomach and duodenum but also due to suppression of appetite by stimulation of anorexigenic and inhibition of orexigenic hormones of the gut-brain axis. However, it also results in malabsorption of several vitamins and minerals such as B2, B3, B6, folate, B12, Vitamin D, and A, calcium, copper, and zinc, which are essential for homeostasis [3]. Hence, supplementation of these vitamins is vital to avoid complications resulting from their deficiencies. It is unknown why certain individuals, despite supplement use, proceed to develop mineral and vitamin deficiencies while others do not. Furthermore, there is an unpredictable nature on why some patients clinically deteriorate more than others after an RYGBS. Here we present the case of a 45-year-old female patient who presented with various micronutrient deficiencies after RYGBS.

## Case presentation

A 45-year-old female with a past medical history significant for rheumatic mitral valve disease with mechanical mitral valve replacement, on warfarin, grade 3 obesity status post-RYGBS one year ago, presented to our hospital with profound weakness, nausea, vomiting, diarrhea, and inability to tolerate any oral intake. She lost over 200 pounds in six months and was so debilitated that she became bedridden. After her original surgery, she developed an anastomotic stricture for which her bariatric surgeon did a dilatation, but she was lost to follow up after the surgeon moved out to a different city. Upon initial evaluation in the Emergency Department, the patient was hypotensive with a blood pressure of 80/58 millimeters of mercury (mmHg), tachycardic with a heart rate of 115 beats per minute (bpm), and slightly tachypneic with a respiratory rate of 15 breaths per minute saturating well on room air. Her hypotension was unresponsive to fluids, and she had to be transferred to the Intensive Care Unit for pressor support and colloid infusion. On exam, the patient was severely cachexia and sarcopenic with diffuse anasarca. Upon examination of the skin, she was noted to have erythematous pruritic macules and papules concerning dermatitis along with follicular hyperkeratosis and alopecia. A grade 2 sacral decubitus ulcer was present. A neurologic exam showed symmetric proximal and distal muscle atrophy in the upper and lower extremities, hypotonia, and significantly decreased power in lower extremity flexors and extensors along with hyporeflexia. She was noted to have numbness and paresthesia along with loss of proprioception. Significant laboratory abnormalities include microcytic anemia with a hemoglobin of 10.7 grams per deciliter (g/dl) and leucopenia with a white blood cell count of 3,000 per cubic milliliter (K/uL). The chemistry panel revealed a potassium of 3.2 millimole per liter (mmol/L), calcium of 7.2 milligrams per deciliter (mg/dl), albumin of 1.6 grams per deciliter (g/dl) with a total protein of 4.4 g/dl along with mildly elevated transaminases including alanine transaminase of 64 units per liter (U/L), aspartate transaminase of 123 U/L and alkaline phosphatase at 167 U/L. Prothrombin time (PT) was elevated at 86.8 seconds and the international normalized ratio (INR) was elevated at 8.1 (Table 1). Further, workup for micronutrient deficiencies revealed abnormally low levels of copper at 31.5 micrograms per deciliter (ug/dl), thiamine 66 nanomoles per liter (nmol/L), B6 9.3 nmol/L, zinc at 35 ug/dl and vitamin A at 0.11 milligrams per liter (mg/L). Surprisingly, the patient’s B12 level was elevated at 2,552 picograms per milliliter (pg/ml). Magnesium and phosphorus were within normal limits (Table 2).

**Table 1:** Hematology Lab Findings PT: Prothrombin time; INR: International normalized ratio; TSH: Thyroid-stimulating hormone; RDW: Red blood cell distribution width.

Labs	Patient's Labs	Reference Levels
WBC	3.5	3.5-11 k/uL
RBC	1.85	3.79-5.11 M/uL
Hemoglobin	6.3	11.7-15 g/dL
Hematocrit	19	35-46%
RDW	17.7	11.5-15%
Neutrophils	2.15	1.6-8.3 K/uL
Lymphocytes	0.97	0.7-4.0 K/uL
PT	82.3	9.0-12.5 seconds
PT-INR	8.1	0.8-1.2
TSH	3.38	0.34-5.60 uIU/mL

**Table 2:** Chemistry and Vitamin Levels BUN: Blood urea nitrogen; eGFR: estimated glomerular filtration rate; ALT: Alanine transaminase; AST: Aspartate transaminase

Labs	Patient's labs	Reference Levels
Sodium	139	136-146 mmol/L
Potassium	3.9	3.5-5.1 mmol/L
Chloride	106	96-102 mmol/L
Co2	22	19-32 mmol/L
BUN	64	6-23 mg/dL
Creatinine	1.5	0.5-1.3 mg/dL
Calcium	7	8.4-10.5 mg/dL
Anion gap	11	2-11 mmol/L
eGFR	44	90-120 ml/min/m2
Total protein	3.5	6.6-8.7 g/dL
Albumin	1.5	3.5-5.2 g/dL
Globulin	2.9	2.3-4.9 g/dL
ALT	26	0-32 U/L
AST	32	0-31 U/L
Bilirubin	2.2	0.3-1.2 g/dL
Magnesium	2.7	1.6-2.6 mg/dL
Vitamin A	0.11	0.3-1.2 mg/L
Vitamin B1	48	70-180 nmol/L
Vitamin B12	1635	211-911 pg/mL
Vitamin B6	9.3	20-125 nmol/L

**Figure 1:**
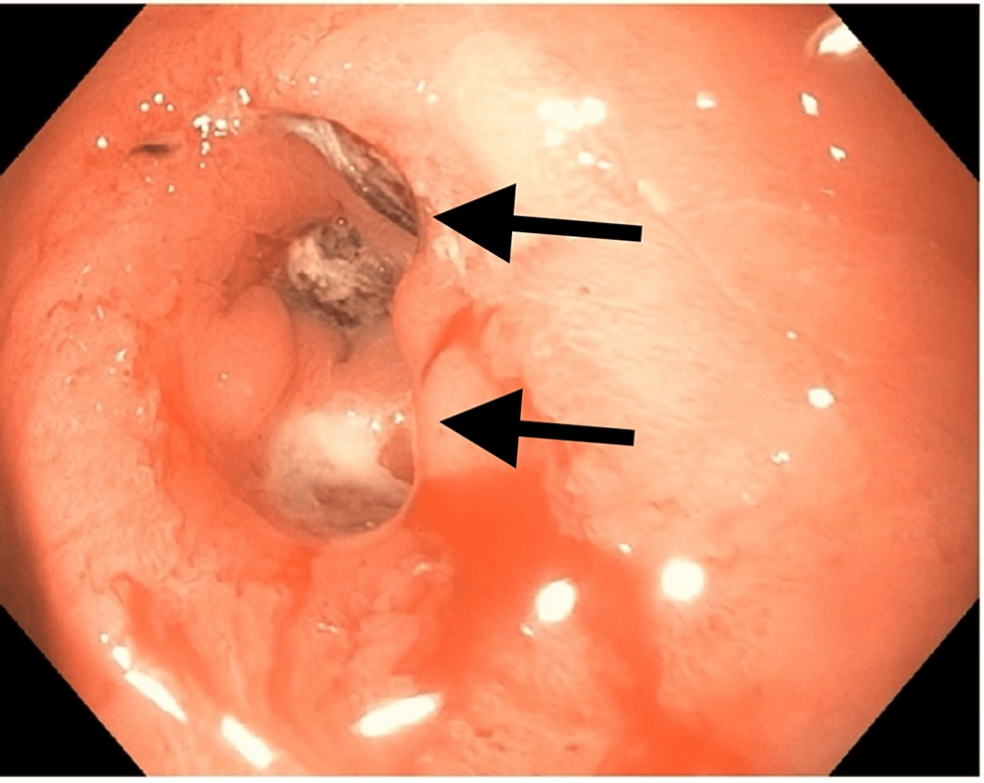
EGD illustrating the 10 mm stricture (top arrow) and ulcer (bottom arrow)

Over the next few days, the patient was started on parental nutrition administered via a peripherally inserted central catheter (PICC) line at a rate of 72 ml/hr which resulted in an improvement in her hemodynamics. Gastroenterology (GI) was consulted and an Esophagogastroduodenoscopy (EGD) was performed on hospital day 3, where a small clean-based ulcer and a 10-mm anastomotic stricture (Figure 1) were noted. General surgery was consulted at this time for concern that the patient would need serial dilations of strictures and possible reversal of the bypass procedure. The team concluded that a revision of the gastrojejunostomy would be difficult in her malnourished state, and the patient was started on Total Parenteral Nutrition (TPN). The patient’s appetite and energy levels improved on TPN, and she was able to participate in physical rehabilitation. A repeat EGD done on hospital day 7 revealed a patent anastomosis without strictures. The patient started tolerating oral intake and subsequently, TPN was discontinued. She was discharged home with instructions to follow up with a bariatric surgeon for the reversal of her RYGB.

## Discussion

Four types of bariatric surgeries are performed for weight loss in modern medicine [4]: adjustable gastric banding, sleeve gastrectomy, Roux-en-Y gastric bypass, and biliopancreatic diversion [5]. Of these, sleeve gastrectomy is the most common bariatric procedure performed in the US, with less morbidity compared to RYGBS and biliopancreatic diversion. RYGBS is the oldest, first being described in 1966 [4]. Traditionally, this has been a mainstay of bariatric procedures, however, in recent times this procedure lost popularity due to several associated serious complications and the emergence of safer and less invasive procedures. Common complications include anastomotic strictures, marginal ulceration, and clinically significant vitamin deficiencies [6]. Anastomotic strictures typically present weeks to months post-operatively and are an early complication of this procedure with a reported 3-7% incidence rate in most literature [6]. Marginal ulceration occurs in 1-16% of patients postoperatively, and 50% of patients with this complication present within the first year after surgery. Gastro-gastric fistulas and small bowel obstruction are more life-threatening complications, which typically take years to develop. Dumping syndrome is commonly seen in female patients and can be an early or late manifestation several years after surgery. Micronutrient deficiency is extremely common after RYGB, and routine vitamin supplementation is required for all patients postoperatively. The literature suggests that the most common micronutrient deficiencies are iron, calcium, vitamin D, and B12. However, there is a variable range of nutritional deficiencies in various studies. For instance, a study in Norway during 2018-2020 evaluated approximately 490 patients roughly 11-12 years post-Roux-en-Y [7]. Out of the 490 patients, 361 patients reported medication adherence. Of these 361 patients, folate deficiency was found in 11%, vitamin B2 in 29%, and vitamin B6 in 17%. In those that were non-adherent, the deficiencies rose to 34% in folate, and 52% in both vitamin B2 and vitamin B6. Additionally, 95% reported adherence to vitamin B12 supplements, yet 16% had sub-optimal levels. Furthermore, sub-optimal vitamin D levels were found in 52% of people who were adherent to calcium/vitamin D supplements and increased to 78% in non-adherent individuals [7]. Another study evaluated 169 patients and evaluated the patients six months, one year, two years, three years, and four years post-Roux-en-Y [3]. At four years, 33% were anemic, 38% iron deficient, 20% calcium deficient, 20% zinc deficient, 29% vitamin D deficient and 13% vitamin B12 deficient. This study also showed that the most common deficiencies are iron, vitamin D, calcium, and vitamin B12 [3]. However, compared to the first study, zinc deficiency was also found to be relatively common.

Various clinical manifestations of these micronutrient deficiencies can be observed in post-operative Roux-en-Y patients. Calcium deficiency leads to muscle cramps, paresthesia, hyperreflexia, tetany, and even seizures. Chvostek and trousseau signs can be elicited in these patients. Vitamin D deficiency will lead to the same downstream effects due to hypocalcemia. Furthermore, this can lead to osteomalacia in adults. Vitamin B1, and thiamine, deficits can lead to dilated cardiomyopathy and high-output heart failure (wet beriberi). Furthermore, B1 deficiency can cause Wernicke encephalopathy acutely and eventually, progress to Korsakoff psychosis. Vitamin B2 serves as an important cofactor for a variety of redox reactions and deficiency can lead to cheilosis, glossitis, and corneal vascularization. Vitamin B6, pyridoxine, deficiency will lead to peripheral neuropathy and sideroblastic anemia. Furthermore, patients can also clinically present with glossitis, seborrheic dermatitis, hyperirritability, and seizures. Vitamin B9 (folate) and Vitamin B12 (cobalamin) deficiencies lead to hyper-segmented neutrophils, glossitis, and macrocytic anemia. Vitamin B9 deficiency in pregnant patients will lead to neural tube defects in offspring while B12 deficiency can lead to paresthesia and irreversible neurological damage like subacute combined degeneration. Zinc deficiency can cause delayed wound healing, hypogonadism, hair loss, perioral rash, dysgeusia, and anosmia. Lastly, vitamin A deficiency can lead to night blindness, Bitot spots, xerosis cutis (dry skin), and immunosuppression. Our patient manifested peripheral edema, pellagra, follicular hyperkeratosis of the tongue, and the loss of hair and teeth and labs indicated a deficiency in vitamin A, B1, vitamin D, zinc, and copper. Practicing physicians should consider the possible nutritional consequences of RYGBS and prophylactic vitamins and nutritional supplements should be considered in all patients. It is recommended to take multivitamins twice a day (chewable during the first 3-6 months) [8]. This multivitamin should include at least 12 mg of Thiamine, 400 mcg of folic acid, and 18 mg of iron. It is advised to obtain at least 1,200-1,500 mg of calcium which should be divided into multiple doses each day. It is recommended to receive roughly 2,000-3,000 units of vitamin D titrated to target a serum 25-hydroxyvitamin D level of >30 ng/mL [8]. Patients should receive 8-22 mg of zinc, 2 mg of copper, and vitamin B12 should be individually titrated to reach the optimal level (500-1300 pg/ml) [8]. Even with nutritional supplements, some patients may continue to develop nutritional deficits. It is important to consistently monitor a patient’s post-op long-term. If a patient deteriorates clinically due to the procedure, it may be worth considering the reversal of the surgery. Lastly, further research is needed to explore why certain individuals are more vulnerable to nutritional deprivation and other major complications than others.

## Conclusions

This case outlines an unfortunate patient who developed several mechanical complications as well as micronutrient deficiencies of RYGB. Several key takeaways from this case should be implemented in the practice of managing a patient with RYGB, including the importance of follow-up in these patients, as they are at high risk for vitamin deficiencies and severe malnutrition. As we saw with this patient, there was no true post-operative recovery and it spiraled to the point where she was bedridden. Patients considering this procedure need to be thoroughly educated. Next, the follow-up must happen with a surgeon who has originally done the procedure, whenever possible, as familiarity of anatomy would aid in prompt consideration of reversal when needed. Finally, this elective surgery should only be performed when the patient has failed other treatment options. With the availability of alternate medical and surgical modalities for weight loss, such as Glucagon-Like Peptide -1 (GLP-1) agonists and sleeve gastrectomy, the side effects of bypass procedures typically outweigh the benefits so this should be reserved for special circumstances.
